# Efficacy of Human Albumin Infusion in Advanced Cirrhosis and Acute-on-Chronic Liver Failure: Implications for Renal Protection and Oncologic Outcomes

**DOI:** 10.7759/cureus.93157

**Published:** 2025-09-24

**Authors:** Adeel Ahmed, Shivam Singla, Bhavna Singla, Sunita Kumawat, FNU Muskan, FNU Komal, Lachhmi Bai, Prem Chand, Shabir Khan

**Affiliations:** 1 Internal Medicine, District Headquarter Hospital Teaching Hospital, Gujranwala, PAK; 2 Internal Medicine, TidalHealth Peninsula Regional, Salisbury, USA; 3 Internal Medicine, Erie County Medical Center Health Campus, Buffalo, USA; 4 Internal Medicine, Hackensack Meridian Ocean Medical Center, New Jersey, USA; 5 Internal Medicine, Jinnah Sindh Medical University, Karachi, PAK; 6 Internal Medicine, Jinnah Postgraduate Medical Center, Karachi, PAK; 7 Internal Medicine, Chandka Medical College, Larkana, PAK; 8 Internal Medicine, City Medical Centre, Kandhkot, PAK; 9 Internal Medicine, Combined Military Hospital Lahore Medical and Dental College, Lahore, PAK

**Keywords:** acute-on-chronic liver failure, albumin infusion, cirrhosis, hepatorenal syndrome, refractory ascites, renal outcomes, survival, systematic review

## Abstract

This systematic review evaluated the role of human albumin infusion in patients with decompensated cirrhosis, refractory ascites, acute-on-chronic liver failure (ACLF), and hepatorenal syndrome type 1 (HRS-1). A total of 398 studies were screened, with 125 excluded for not meeting eligibility criteria, and the final selection comprised randomized controlled trials and clinically relevant post hoc analyses published between 2016 and 2024. The findings suggest that albumin infusion confers significant benefits in reducing the incidence of post-paracentesis circulatory dysfunction (PICD), acute kidney injury (AKI), and hyponatremia, while also improving survival in selected patient populations. Comparative trials demonstrated that albumin may outperform standard care and support vasoconstrictor-based regimens, though cost-effectiveness concerns remain when compared to alternatives such as midodrine. The evidence base is strengthened by large multicenter trials with objective outcomes, though limitations include small pilot studies, post hoc subgroup analyses, and heterogeneity in dosing strategies. Overall, albumin infusion appears to be a clinically valuable intervention in the management of advanced cirrhosis, but further trials are warranted to clarify its long-term effects, particularly on hepatocellular carcinoma progression and optimal treatment regimens.

## Introduction and background

Decompensated cirrhosis represents the advanced stage of chronic liver disease, characterized by complications such as ascites, variceal bleeding, hepatic encephalopathy, and renal dysfunction [1]. Among these, acute kidney injury (AKI), particularly in the form of hepatorenal syndrome (HRS), is a frequent and devastating complication associated with high morbidity and mortality. The hemodynamic derangements in cirrhosis-systemic vasodilation, effective hypovolemia, and reduced renal perfusion-play a central role in the pathogenesis of AKI [2]. Early preventive strategies to preserve renal function are therefore crucial in improving outcomes in this patient population.

Albumin, a plasma-derived protein with both oncotic and non-oncotic properties, has long been employed in cirrhosis management. Beyond its role in volume expansion, albumin exerts pleiotropic effects, including antioxidant activity, immunomodulation, and endothelial stabilization, which may mitigate circulatory dysfunction and renal impairment [3,4]. Long-term albumin administration has shown promise in reducing complications of cirrhosis, including infections and renal dysfunction, while short-term use is standard in specific contexts such as large-volume paracentesis, spontaneous bacterial peritonitis, and HRS [5].

Another important concern in cirrhosis is the progression to hepatocellular carcinoma (HCC). Inflammatory signaling, oxidative stress, and persistent hepatocellular injury in decompensated cirrhosis create a microenvironment conducive to malignant transformation [6]. Albumin, through its capacity to scavenge free radicals and bind toxic metabolites, may theoretically delay disease progression and influence carcinogenesis, though clinical evidence remains limited. Understanding whether albumin infusion not only prevents renal injury but also impacts HCC progression is a critical area of ongoing investigation [7].

This systematic review aims to evaluate the efficacy of albumin infusion in preventing acute kidney injury and delaying HCC progression among patients with decompensated cirrhosis. By synthesizing evidence from randomized controlled trials and clinical studies, the review seeks to clarify the role of albumin as a therapeutic intervention, highlight its impact on survival and long-term outcomes, and identify gaps for future research.

## Review

Materials and methods

Study Design and Protocol

This systematic review was conducted in accordance with the Preferred Reporting Items for Systematic Reviews and Meta-Analyses (PRISMA) 2020 guidelines [8]. The protocol was designed to follow the Population, Intervention, Comparator, and Outcomes (PICO) framework, ensuring a structured approach to evidence identification and synthesis [9].

Eligibility Criteria

Studies were included if they were randomized controlled trials (RCTs) or clinical trials evaluating the role of human albumin infusion in patients with decompensated cirrhosis, acute-on-chronic liver failure (ACLF), refractory ascites, or hepatorenal syndrome type 1 (HRS-1). Eligible studies compared albumin infusion either to standard medical therapy, placebo, or alternative pharmacological interventions such as midodrine or terlipressin. Outcomes of interest included survival, renal function, incidence of cirrhosis-related complications, and hospitalizations. Studies published in English between 2016 and 2024 were considered for inclusion. Post hoc analyses were included if they provided clinically relevant primary outcome data. Case reports, reviews, editorials, and non-comparative observational studies were excluded.

Information Sources and Search Strategy

A comprehensive literature search was carried out across multiple databases, including PubMed/MEDLINE, Embase, Cochrane Central Register of Controlled Trials (CENTRAL), and Web of Science. The search strategy combined controlled vocabulary and free-text terms related to “albumin,” “cirrhosis,” “ascites,” “hepatorenal syndrome,” and “acute-on-chronic liver failure.” Reference lists of included articles were screened manually to identify additional eligible studies. The final search was conducted in July 2025, ensuring that the most recent evidence was incorporated.

Study Selection and Data Extraction

Two independent reviewers screened titles and abstracts, followed by a full-text review of potentially eligible studies. Discrepancies were resolved by discussion or consultation with a third reviewer. A standardized extraction form was used to collect data on study design, population characteristics, sample size, interventions, comparators, outcomes, and key findings.

Risk of Bias and Quality Assessment

The methodological quality of included RCTs was assessed using the Cochrane Risk of Bias 2.0 tool [10], evaluating domains such as randomization, allocation concealment, blinding, outcome assessment, and selective reporting. Each study was classified as low, high, or having some concerns regarding bias. The overall certainty of evidence for major outcomes was considered in light of study limitations, consistency, and applicability.

Data Synthesis

Due to heterogeneity in interventions, dosing regimens, comparators, and measured outcomes, a narrative synthesis was undertaken rather than a formal meta-analysis. Findings were summarized by clinical context, including prevention of PICD, management of refractory ascites, and treatment of hepatorenal syndrome. Where possible, comparative insights across interventions were highlighted to inform clinical relevance.

Results

Study Selection Process

**Figure 1:**
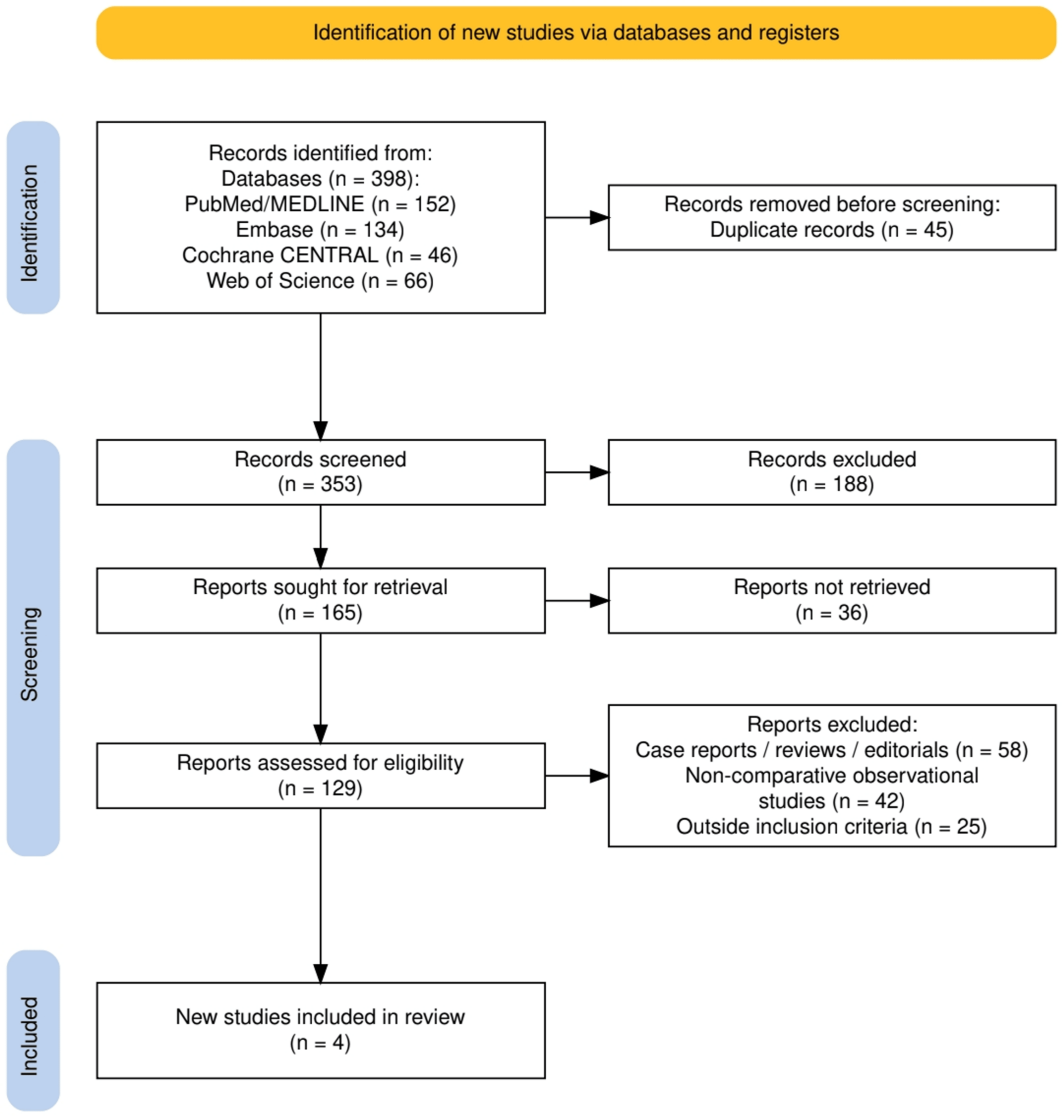
The PRISMA flowchart represents the study selection process. PRISMA: Preferred Reporting Items for Systematic Reviews and Meta-Analyses

The flow of studies is summarized in Figure 1, which outlines the PRISMA-based selection process. A total of 398 records were initially identified across four databases: PubMed/MEDLINE (n = 152), Embase (n = 134), Cochrane CENTRAL (n = 46), and Web of Science (n = 66). After removing 45 duplicates, 353 records were screened, of which 188 were excluded at this stage. From the remaining 165 reports sought for retrieval, 36 could not be obtained, leaving 129 full-text articles for eligibility assessment. Following detailed evaluation, 58 case reports/reviews/editorials, 42 non-comparative observational studies, and 25 studies outside the inclusion criteria were excluded. Ultimately, four randomized or clinical trials met the eligibility requirements and were included in the final review.

Characteristics of the Selected Studies

The selected studies, summarized in Table 1, were all RCTs conducted between 2016 and 2024, focusing on patients with decompensated cirrhosis, refractory ascites, ACLF, or hepatorenal syndrome. Sample sizes ranged from 75 to 196 participants, with interventions primarily involving albumin infusion either alone or in combination with standard medical therapy or vasoconstrictors, compared against placebo, no albumin, or alternative agents such as midodrine. Outcomes assessed included survival, renal function, incidence of PICD, hepatic encephalopathy, hyponatremia, and hospital utilization. Overall, albumin was associated with improved survival, reduced renal dysfunction, and fewer cirrhosis-related complications in several trials, though results varied depending on the comparator, with some studies highlighting cost-effectiveness concerns and potential adverse events when albumin was combined with vasoconstrictors.

**Table 1:** Summary of randomized controlled trials assessing albumin infusion in advanced cirrhosis and its complications. RCT: Randomized controlled trial; ACLF: Acute-on-chronic liver failure; PICD: Paracentesis-induced circulatory dysfunction; AKI: Acute kidney injury; SMT: Standard medical treatment; HRS-1: Hepatorenal syndrome type 1; SCr: Serum creatinine

Author/Year	Study Design	Population	Sample Size (n)	Intervention	Comparator	Outcomes Measured	Key Findings
Pompili et al., 2024 (ANSWER trial, post hoc) [11]	RCT, post hoc analysis	Decompensated cirrhosis + insulin-treated type 2 diabetes mellitus, with uncomplicated ascites	85	Long-term human albumin (HA) + standard medical treatment (SMT)	SMT alone	Overall survival, incidence of paracenteses, hepatic encephalopathy, bacterial infections, renal dysfunction, electrolyte disorders, hospital admissions, and hospital stay	Albumin group had higher survival (86% vs 57%, p=0.016), fewer cirrhosis-related complications, and fewer hospital days; admissions were similar.
Arora et al., 2020 [12]	RCT	ACLF patients undergoing modest-volume paracentesis (<5 L)	80 (40 albumin, 40 no albumin)	Albumin infusion (8 g/L ascitic fluid removed)	No albumin	PICD incidence, hepatic encephalopathy, hyponatremia, AKI, mortality, plasma renin activity	Albumin reduced PICD (30% vs 70%), AKI (30% vs 62.5%), hyponatremia, encephalopathy, and mortality (27.5% vs 62.5%)
Yosry et al., 2019 [13]	Pilot RCT	Cirrhotic patients with refractory ascites undergoing large-volume paracentesis (LVP)	75	Albumin infusion post-LVP	Oral midodrine (2 days or 30 days)	Renal impairment, hyponatremia, systemic and portal hemodynamics, mortality, urine sodium excretion, and cost	No difference between groups in renal impairment, hyponatremia, or mortality at 6 and 30 days; long-term midodrine improved renal perfusion and sodium excretion; midodrine is significantly cheaper
Boyer et al., 2016 (REVERSE trial) [14]	Phase 3 RCT	Adults with cirrhosis, ascites, and HRS-1	196 (97 terlipressin + albumin, 99 placebo + albumin)	IV terlipressin + albumin	Placebo + albumin	Confirmed HRS reversal (CHRSR), HRS reversal, serum creatinine change, transplant-free survival, overall survival	Terlipressin + albumin led to greater SCr reduction (-1.1 vs -0.6 mg/dL, p<0.001) and higher HRS reversal rates (23.7% vs 15.2%, NS); overall survival similar; ischemic events higher in terlipressin group

Risk of Bias Assessment

The risk of bias assessment, summarized in Table 2, indicates that the majority of the included randomized trials demonstrated low risk across most domains, particularly regarding randomization, adherence to interventions, completeness of outcome data, and objective measurement of endpoints such as survival and renal function. Larger multicenter trials showed robust methodological quality with predefined endpoints and trial registration, strengthening the validity of their findings. However, some concerns were noted in post hoc and pilot studies, primarily due to the inherent limitations of subgroup analyses, smaller sample sizes, and limited reporting, which may introduce selective reporting bias. Overall, while the evidence base is largely reliable, interpretation should be cautious when drawing conclusions from studies with methodological concerns.

**Table 2:** Risk of bias assessment of included randomized controlled trials on albumin infusion in advanced cirrhosis RCT: Randomized controlled trial; SCr: Serum creatinine

Study	Randomization Process	Deviations from Intended Interventions	Missing Outcome Data	Measurement of the Outcome	Selective Reporting	Overall Risk of Bias
Pompili et al., 2024 (ANSWER trial, post hoc) [11]	Low (original ANSWER trial was randomized, though this is post hoc subgroup)	Some concerns (post hoc analysis introduces risk of bias)	Low (complete follow-up in subgroup reported)	Low (objective outcomes like survival, renal dysfunction)	Some concerns (not primary trial objective)	Some concerns
Arora et al., 2020 [12]	Low (randomized with comparable baseline characteristics)	Low (albumin vs no albumin, protocol adhered)	Low (adequate reporting of 80 patients)	Low (hard clinical outcomes: AKI, mortality)	Low (outcomes prespecified, NCT registered)	Low risk
Yosry et al., 2019 [13]	Some concerns (pilot RCT, small sample, unclear randomization method reported)	Low (intervention delivered as planned)	Some concerns (possible attrition bias not clearly addressed)	Low (objective renal outcomes and mortality)	Some concerns (pilot nature, limited reporting)	Some concerns
Boyer et al., 2016 (REVERSE trial) [14]	Low (well-designed multicenter RCT)	Low (blinding with placebo + albumin)	Low (196 patients adequately followed)	Low (objective SCr, survival outcomes)	Low (registered, predefined endpoints)	Low risk

Discussion

Across the selected trials, albumin infusion consistently demonstrated benefits in reducing complications of decompensated cirrhosis. In the ANSWER post hoc analysis (Pompili et al., 2024 [11]), long-term albumin significantly improved survival and reduced cirrhosis-related complications compared with standard care. Arora et al. showed that albumin markedly decreased the risk of PICD, AKI, and mortality in ACLF, even after modest-volume paracentesis [12]. Yosry et al. found that albumin and oral midodrine had comparable efficacy for preventing renal impairment and hyponatremia post-paracentesis, though midodrine offered cost advantages [13]. In contrast, the REVERSE trial (Boyer et al.) highlighted that albumin alone had a limited impact on hepatorenal syndrome type 1 (HRS-1), whereas combining it with terlipressin yielded greater improvements in renal function, though without a survival benefit [14]. Collectively, these findings emphasize albumin’s role in renal protection and complication reduction, while also underscoring the value of adjunctive or alternative therapies.

The protective effect of albumin in cirrhosis can be explained by both its oncotic and non-oncotic properties [15]. By expanding plasma volume, albumin reduces the hemodynamic derangements that predispose patients to AKI and circulatory dysfunction after paracentesis. Furthermore, albumin binds toxins, modulates inflammatory responses, and stabilizes endothelial function, all of which are relevant in decompensated cirrhosis and ACLF, where systemic inflammation drives organ failure [16,17]. The consistent reduction in PICD and renal dysfunction seen in trials such as Arora et al. supports its frontline use after paracentesis [12]. At the same time, data from Yosry et al. suggest that cost-effective vasoconstrictor strategies like midodrine may be suitable alternatives, particularly in low-resource settings [13]. Importantly, in severe cases like HRS-1, albumin alone appears insufficient, but its synergistic role alongside vasoconstrictors such as terlipressin makes it a cornerstone of therapy [18]. This highlights that albumin should not be viewed as a universal solution but rather as an essential component of tailored management strategies for cirrhosis.

These findings are broadly consistent with current international guidelines. Both the EASL and AASLD recommend albumin for the prevention of PICD after large-volume paracentesis and in combination with vasoconstrictors for HRS treatment [19,20]. Meta-analyses have similarly confirmed albumin’s role in reducing hyponatremia, renal dysfunction, and mortality in selected patient populations. However, the post hoc evidence from the ANSWER trial (Pompili et al.) adds nuance by demonstrating long-term benefits of albumin beyond renal outcomes, including improved survival and fewer complications in patients with comorbid diabetes [11]. The results of Yosry et al. differ slightly from guideline consensus by suggesting non-inferiority of midodrine compared to albumin, reflecting population-specific differences and the potential influence of treatment duration [13]. The REVERSE trial’s findings of improved renal function but no survival advantage align with the broader recognition that HRS-1 remains difficult to reverse without liver transplantation [14]. Overall, the evidence base reinforces guideline recommendations while also pointing to evolving strategies that integrate albumin with adjunct therapies.

A major strength of the included evidence is that most studies were RCTs, which reduces bias and allows direct comparisons between albumin and alternative strategies. Objective and clinically relevant endpoints such as renal impairment, survival, and cirrhosis-related complications were consistently reported across studies. Large multicenter designs, as seen in the REVERSE trial, enhance generalizability across different patient populations [14]. Importantly, there was a degree of consistency across trials: albumin use was repeatedly associated with reduced risk of AKI, hyponatremia, and paracentesis-induced circulatory dysfunction (PICD), reinforcing its role as a cornerstone in managing decompensated cirrhosis [21].

Despite these strengths, several limitations must be acknowledged. The ANSWER post hoc analysis (Pompili et al.), while suggestive of survival benefit, is inherently limited by its retrospective nature and restricted subgroup focus [11]. The Yosry et al. pilot trial had a relatively small sample size, limiting its statistical power to detect differences in outcomes such as mortality [13]. Considerable heterogeneity in interventions was also noted, including variable albumin dosing regimens, treatment durations, and combinations with vasoconstrictors, making direct comparisons difficult. Furthermore, none of the included trials reported on long-term outcomes such as HCC progression, which was a key motivation for this review. From a methodological standpoint, our review itself may be subject to publication bias, language restrictions, and the constraints of the literature search cut-off date, which could limit comprehensiveness.

An additional dimension worth noting is the potential oncologic impact of albumin therapy. Patients with decompensated cirrhosis remain at high risk for HCC due to persistent oxidative stress, systemic inflammation, and impaired hepatic regeneration. Albumin, beyond its hemodynamic benefits, possesses antioxidant and immunomodulatory properties that may theoretically mitigate carcinogenic pathways. While none of the included trials assessed HCC progression as an endpoint, this represents an important gap in the literature. Future long-term studies should explore whether sustained albumin administration can influence hepatocarcinogenesis or cancer-related survival, potentially extending its role beyond renal protection into oncology prevention strategies.

The findings collectively support the continued use of albumin infusion after large-volume paracentesis to reduce the risk of PICD, AKI, and related complications, particularly in patients with ACLF or decompensated cirrhosis [22]. Evidence from Arora et al. strengthens the case for albumin as standard of care in this setting [12]. However, the cost-effectiveness issue highlighted by Yosry et al. cannot be overlooked, especially in resource-limited settings where alternatives such as oral midodrine may provide comparable short-term outcomes [13]. In HRS-1, albumin alone is insufficient, but in combination with terlipressin, it remains the best-supported therapy, consistent with EASL and AASLD guidelines [23]. Overall, our review reinforces current recommendations but also suggests a need for personalized approaches, balancing efficacy, safety, and cost considerations.

There are several critical gaps in the evidence base. Long-term outcomes such as HCC progression and overall survival beyond 1-2 years remain poorly studied, despite their central importance in cirrhosis care. The optimal dosing strategy, duration, and patient selection criteria for albumin therapy are yet to be standardized across trials. Comparative effectiveness research is needed to determine whether albumin or vasoconstrictors like midodrine and terlipressin should be prioritized in different clinical scenarios [24]. Additionally, cost-effectiveness studies are urgently required, especially for low- and middle-income countries where albumin may be prohibitively expensive. Future research should focus on large-scale, multicenter RCTs and updated meta-analyses that address these knowledge gaps and integrate patient-centered outcomes.

## Conclusions

Our systematic review suggests that albumin infusion provides clear benefits in preventing AKI, reducing PICD, and improving short-term survival in decompensated cirrhosis and ACLF, with synergistic effects when combined with vasoconstrictors in HRS-1. However, the evidence remains heterogeneous, with limitations in trial design, sample size, and long-term outcome reporting. Cost and access issues further complicate universal adoption. Moving forward, robust RCTs with long-term endpoints, including HCC progression, are essential to define the full role of albumin in chronic liver disease. Until then, albumin should remain a key but tailored component of cirrhosis management, guided by clinical context and resource availability.
